# Tailoring recombinant lipases: keeping the His-tag favors esterification reactions, removing it favors hydrolysis reactions

**DOI:** 10.1038/s41598-018-27579-8

**Published:** 2018-07-03

**Authors:** Janaina Marques de Almeida, Vivian Rotuno Moure, Marcelo Müller-Santos, Emanuel Maltempi de Souza, Fábio Oliveira Pedrosa, David Alexander Mitchell, Nadia Krieger

**Affiliations:** 1 Departamento de Bioquímica e Biologia Molecular, Universidade Federal do Paraná, Cx.P. 19046 Centro Politécnico, Curitiba, 81531-980 Paraná, Brazil; 2 Departamento de Química, Universidade Federal do Paraná, Cx.P. 19081 Centro Politécnico, Curitiba, 81531-980 Paraná, Brazil

## Abstract

We determined the effect of the His-tag on the structure, activity, stability and immobilization of LipC12, a highly active lipase from a metagenomic library. We purified LipC12 with a N-terminal His-tag and then removed the tag using tobacco etch virus (TEV) protease. Circular dichroism analysis showed that the overall structure of LipC12 was largely unaffected by His-tag removal. The specific hydrolytic activities against natural and artificial substrates were significantly increased by the removal of the His-tag. On the other hand, His-tagged LipC12 was significantly more active and stable in the presence of polar organic solvents than untagged LipC12. The immobilization efficiency on Immobead 150 was 100% for both forms of LipC12 and protein desorption studies confirmed that the His-tag does not participate in the covalent binding of the enzyme. In the case of immobilized LipC12, the His-tag negatively influenced the hydrolytic activity, as it had for the free lipase, however, it positively influenced the esterification activity. These results raise the possibility of tailoring recombinant lipases for different applications, where the His-tag may be retained or removed, as appropriate for the desired activity.

## Introduction

Affinity tags have gained importance over the last decades as a purification tool for recombinant proteins^1^. Such tags include arg-tags, the calmodulin-binding peptide, cellulose-binding domains, DsbA, the c-myc-tag, glutathione S-transferase, the FLAG-tag, the HAT-tag, the maltose-binding protein, NusA, the S-tag, the SBP-tag, the Strep-tag, thioredoxin and His-tags^2^. Of these, His-tags (i.e. the polyhistidine affinity tag) are the most used, with approximately 25% of all protein structures in the Protein Data Bank (PDB) having been obtained for His-tagged proteins^3^. His-tagged proteins are purified by metal affinity chromatography, since the imidazole ring of the histidine establishes coordination bonds with immobilized ions (such as Co^2+^, Ni^2+^, Cu^2+^ or Zn^2+^)^2^. His-tags can be used for purifying proteins, under either non-denaturing or denaturing conditions, and the recombinant proteins can be eluted under mild conditions^4,5^. In addition to simplifying purification, His-tags are designed to optimize protein expression and therefore result in high yields. They also allow the detection of the recombinant protein^6,7^.

His-tags are bulky and might affect the conformation of the enzyme. The polyhistidine region typically consists of six consecutive histidine residues, but it can vary from two to ten histidine residues. In addition, His-tagged heterologous proteins that are expressed in *Escherichia coli* using the pET vector series^8^ contain several other amino acid residues. For example, use of the vector pET28a leads to the addition of a total of 20 residues at the N-terminal, of which only six are histidine residues.

His-tags are often used in the expression of recombinant lipases. Lipases are of great industrial interest due to their diverse biotechnological applications. They are efficient catalysts not only in conventional aqueous media, but also in non-conventional media, such as organic solvents, ionic liquids, supercritical fluids, and eutectic solvents^9,10^. Depending on the medium, they catalyze reactions of hydrolysis, esterification, transesterification, acetylation and lactonization^11^.

Despite the potential that His-tags have to affect conformation and activity, to date, few studies have assessed their influence on the properties of lipases. In one study, the His-tagged *Staphylococcus xylosus* lipase (His-SXL), overexpressed in *E. coli*, had altered kinetic parameters, altered stereo- and regioselectivity and altered thermostability, compared to the native lipase (SXL)^12,13^. In another study, a recombinant *S. aureus* lipase (SAL), expressed in *E. coli* with a His-tag at the N-terminal, had a lower specific activity for the hydrolysis of tributyrin than the native lipase^14^.

In our laboratory, LipC12, a recombinant lipase containing a His-tag at the N-terminal, was originally isolated from a metagenomic library and characterized by Glogauer *et al*.^15^. Later, LipC12 was immobilized on Immobead 150. One immobilized preparation was obtained using the crude extract obtained after expression of the enzyme and cell breakage, while another was obtained using a preparation that had been purified from the crude extract using a nickel affinity column^16,17^. During these immobilization studies, Madalozzo *et al*.^16^ suggested that the presence of the His-tag might contribute to selective immobilization of His-LipC12 from the crude extract onto Immobead 150. Their argument was that, at the pH of 7.5 used for the immobilization, the histidine residues in the tag would be mostly deprotonated (the pKa of the histidine side chains in the His-tag is reported as 6.0)^18^ and this would favor nucleophilic attack by these side chains on the oxirane group of the support. However, they did not undertake studies to confirm that the His-tag was actually involved in immobilization.

In this work, we systematically investigated the effects of the His-tag on the structure, activity, stability and immobilization of LipC12. We used two strategies for the expression and removal of the His-tag from LipC12: (1) the *lipC12* gene was cloned into pET28a for the expression of the His-tag on the N-terminal of the enzyme^15^ and this homolog was compared with the other recombinant form of LipC12, which was produced by cloning the *lipC12* gene into pET29a for the expression of recombinant LipC12 (denominated rLipC12) without the His-tag; (2) the *lipC12* gene was cloned into pTEV5 for the expression of the His-tag at the N-terminal of the enzyme, with the inclusion of a tobacco etch virus (TEV) cleavage site, enabling posterior His-tag removal^19^.

## Results

All results in this section are presented as the mean of triplicate determinations ± standard deviation of the mean.

### Properties of LipC12 expressed with and without His-tag

Although previous reports have suggested that the His-tag negatively affects the hydrolytic activity of lipases^12,13^, it is not a consensus. In this work, we compared the activities and the immobilization behavior of LipC12 expressed with and without the His-tag. In order to do so, the *lipC12* gene was cloned into pET28a( + ) and pET29a( + ), producing CSF-His-LipC12 (crude soluble fraction containing LipC12 with His-tag) and CSF-rLipC12 (crude soluble fraction containing LipC12 without His-tag), respectively, through overexpression in *E. coli* BL21(DE3). In each case, the cell pellet was disrupted by sonication, the crude extract was then centrifuged, and the crude soluble fraction, containing either His-LipC12 or rLipC12, was used for activity and immobilization tests.

The specific hydrolytic activities of these crude soluble fractions were determined in a pHStat against olive oil. The crude soluble fraction containing rLipC12 had a significantly higher specific activity (1938 ± 82 U mg^−1^) than did the crude soluble fraction containing His-LipC12 (1469 ± 63 U mg^−1^) (P < 0.05). This difference in specific hydrolytic activity was not due to differences in protein expression: densitometry analysis of SDS-PAGE gels of the two crude soluble fractions showed that the lipases contributed similar percentages of the overall protein. In the case of His-LipC12, the lipase band (at ~33 kDa) contributed 18.6% ± 0.8% of the overall protein, while in the case of rLipC12, the lipase band (at 31 kDa) contributed 18.0% ± 0.3% (Supplementary Fig. S2).

**Figure 1:**
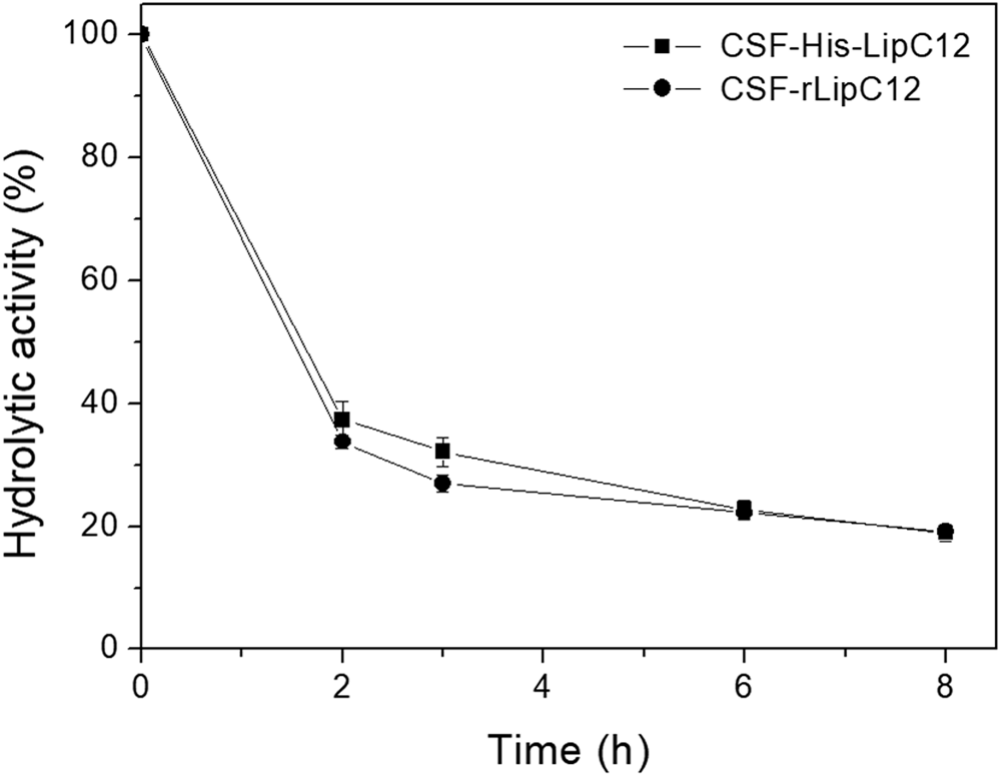
Kinetics of immobilization of CSF-His-LipC12 and CSF-rLipC12 on Immobead 150. The residual hydrolytic activities of the supernatants in aqueous medium were measured against olive oil by the titrimetric method, at 30 °C and pH 7.0.

Subsequently, we investigated the suggestion that the covalent immobilization of CSF-His-LipC12 on the commercial support Immobead 150 involves the His-tag^16^. We used previously established conditions^16^ (i.e. a loading of 200 mg of protein per gram of support) for immobilization of CSF-His-LipC12 and CSF-rLipC12. The kinetics of immobilization were followed by monitoring the residual olive-oil-hydrolyzing activity of the supernatant. The profiles were very similar (Fig. 1), with immobilization efficiencies of 80% being obtained at 8 h for both preparations (Supplementary Table S1). Hereafter, the immobilized preparations are referred to as I-CSF-His-LipC12 and I-CSF-rLipC12.

After immobilization, the olive-oil-hydrolyzing activities of the two LipC12 preparations were determined in the pHStat. As had occurred with the soluble preparations of LipC12, I-CSF-rLipC12 had a significantly higher hydrolytic activity (421 ± 24 U g^−1^ support) than I-His-LipC12 (303 ± 21 U g^−1^ support) (P < 0.05).

Both immobilized preparations were then used to esterify oleic acid with ethanol in *n*-hexane. The esterification activities for both preparations were not significantly different, 66 ± 0 U g^−1^ for I-CSF-rLipC12 and 61 ± 1 U g^−1^ for I-CSF-His-LipC12 (P > 0.05).

Together, these results suggest that the His-tag decreases the hydrolytic activity of both the free and immobilized LipC12. Further studies using purified untagged and His-tagged LipC12 were needed to confirm these results, they are reported in the next subsection.

### Properties of purified LipC12 with and without cleavable His_TEV_-Tag

**Figure 2:**
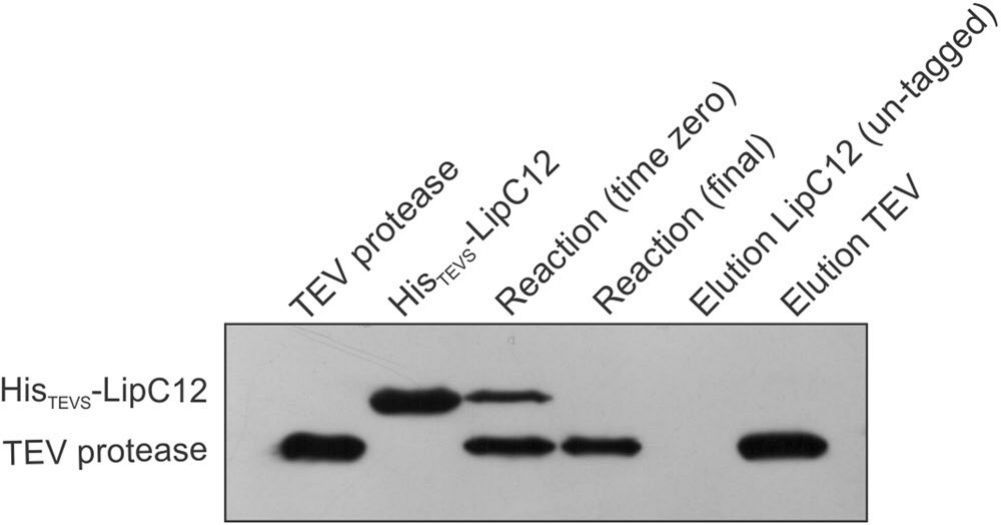
Cleavage of His_TEVs_-LipC12 by TEV protease. The substrate His_TEVS_-LipC12, containing a TEV protease cleavage site, was mixed with the TEV protease at mass ratio 10:1 of substrate:TEV. 1 μg of TEV and 2 μg of other fractions were analyzed by 12% SDS-PAGE followed by western blotting and incubation with Ni-NTA HPR. The full-length blot is reported in Supplementary Figure S6.

We cloned *lipC12* into the vector pTEV5, which introduces the TEV protease cleavage site between the His-tag and the target protein. The resulting LipC12 variant, denominated His_TEVS_-LipC12, was overexpressed in *E. coli* BL21(DE3) and purified using immobilized metal affinity chromatography. The His-tag was later cleaved by TEV protease, with 100% efficiency, as confirmed by western blot (Fig. 2). The cleaved variant was denominated cLipC12. This strategy allowed the cost-effective production of both His_TEVS_-LipC12 and untagged cLipC12, with high purity being obtained in a one-step affinity process (Supplementary Fig. S1).

The *lipC12* gene encodes 293 amino acids and rLipC12 has a theoretical molecular weight of 31.2 kDa. His_TEVS_-LipC12 has 27 additional residues at its N-terminal (MSYYHHHHHHDYDIPTSENLYFQGASH) and has a theoretical molecular weight of 34.5 kDa. After TEV protease cleavage, four of these additional residues (GASH) remain at the N-terminal, such that cLipC12 has a theoretical molecular weight of 31.6 kDa. These theoretical molecular weights were confirmed by SDS-PAGE (Supplementary Fig. 3S).

Several authors have used TEV protease to cleave the His-tag from different target proteins. The amount of TEV required is usually empirically determined, with ratios of target protein to TEV between 100:1 and 5:1 being tested^20^. Our results showed complete cleavage under mild conditions (8 °C for 20 h) using a ratio of 10:1.

### Structural analysis of His_TEVS_-LipC12 and cLipC12 by circular dichroism

**Figure 3:**
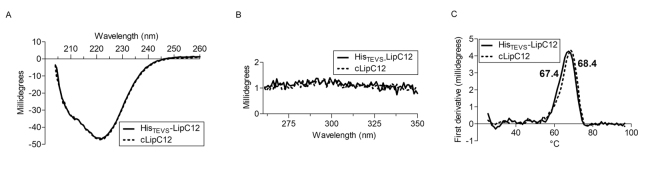
Circular dichroism analysis of His_TEVS_-LipC12 and LipC12. Proteins were diluted to 5 μM in a 10-mM sodium phosphate buffer, pH 7.4, containing 100 mM NaCl. (**A**) CD spectra of His_TEVS_-LipC12 and LipC12 were recorded at 25 °C. (**B**) CD spectra of His_TEVS_-LipC12 and LipC12 in the near-UV (350-250 nm) regions. (**C**) Determination of the melting temperatures (Tm) of His_TEVS_-LipC12 and LipC12. The Tm was calculated by measuring the CD signal at 222 nm with increasing temperature (from 25 to 98 °C) in a continuous ramp mode (1 ^o^C/2 min). The Tm was determined from the first derivative (GraphPrism 6.0).

The far-UV CD spectra of both His_TEVS_-LipC12 and cLipC12 showed typical negative absorption signals of α-helices at about 208 nm and 222 nm, as well as negative absorption signals of antiparallel β-sheets at 215 nm (Fig. 3A). Positive absorption peaks at about 190 nm were not seen because the dynode voltage was above 600 V, resulting in unreliable data^21^. This CD profile is similar to those shown by other lipases^22–25^. The CD spectra for His_TEVS_-LipC12 and cLipC12 overlap completely in the far-UV (250-200 nm) and near-UV (350-250 nm) regions, indicating that the His-tag does not induce structural rearrangements in LipC12. The CD spectra of His_TEVS_-LipC12 and LipC12 were further analyzed by K2D3 online software; for each protein, the estimate was an α-helix content of 36% and a β-sheet content of 20%. There is no previous study in which CD spectra have been used to characterize the structural changes of lipases due to the presence of a His-tag, but CD spectra have been used to show that a His-tag induces structural changes in zinc finger protein ZNF191 (243–368)^26^.

We also compared the thermal stabilities of His_TEVS_-LipC12 and LipC12 by recording CD data at 222 nm to determine the melting temperature (Tm) (Fig. 3B). The Tm decreased by only 1 °C in the presence of the His-tag, which suggests the His-tag has a minimum impact on the thermal stability of LipC12.

### Hydrolytic activity of His_TEVS_-LipC12 and cLipC12

The specific olive-oil-hydrolyzing activities were 2555 ± 17 U mg^−1^ for His_TEVS_-LipC12 and 4590 ± 101 U mg^−1^ for cLipC12. The significantly lower hydrolytic activity in the presence of the His-tag (P < 0.05) confirms the results obtained above, where the hydrolytic activity of CSF-His-LipC12 was lower than that of CSF-rLipC12 and the hydrolytic activity of I-CSF-His-LipC12 was lower than that of I-CSF-rLipC12 (see the first subsection of the results). We also evaluated the activities of His_TEVS_-LipC12 and cLipC12 in the hydrolysis of natural substrates with a range of acyl chain lengths. For all substrates, the activity of His_TEVS_-LipC12 was significantly lower than that of cLipC12 (P < 0.05) (Table 1).

**Table 1:** Relative hydrolytic activities of His_TEVS_-LipC12 and cLipC12 against different triacylglycerols.

Substrate	Relative hydrolytic activities	
	His_TEVS_-LipC12	cLipC12
Olive oil	100 ± 4	173 ± 4
Triolein (C18)	109 ± 5	170 ± 6
Tricaprylin (C8)	188 ± 2	345 ± 9
Tributyrin (C4)	304 ± 8	611 ± 10

Measurements were performed by the titration method using an automatic titrator pHStat. Values represent the mean of triplicates ± standard deviations of the mean.

### Hydrolytic activity of His_TEVS_-LipC12 and cLipC12 against *p*NPD

Under the standard reaction conditions (see methods section) for the hydrolysis of *p*-nitrophenyl decanoate (*p*NPD), cLipC12 had a specific activity of 325 ± 20 U mg^−1^, which is 1.7-fold higher than that of His_TEVS_-LipC12 (192 ± 5 U mg^−1^).

The effect of the *p*NPD concentration on the initial hydrolysis rate was determined for both His_TEVS_-LipC12 and cLipC12 under the standard reaction conditions (Supplementary Fig. S4). The Michaelis-Menten equation was fitted to the data obtained with both enzymes using non-linear regression. In both cases, the coefficient of determination was greater than 0.99. Table 2 shows the parameter values that gave the best fits.

**Table 2:** Apparent kinetic constants for *p*-nitrophenyl decanoate hydrolysis of His_TEVS_-LipC12 and LipC12.

Enzyme	*K*_M_ (mM)	*k*_cat_ (min^−1^)	*k*_cat_/*K*_M_ (mM^−1^ min^−1^)
His_TEVS_-LipC12	1.77 (1.56–2.02)^a^	152 (143–162)	86 (80–92)
cLipC12	1.33 (1.17–1.51)	151 (144–160)	114 (105–123)

^a^The values between parentheses are the 95% confidence intervals.

The *K*_M_ values obtained for His_TEVS_-LipC12 and cLipC12 were different (their 95% confidence intervals did not overlap). On the other hand, the *k*_cat_ values were essentially the same (their 95% confidence intervals overlap almost completely). Due to the difference in the *K*_M_, the values of *k*_cat_/*K*_M_ were also different for the two enzymes, with that for His_TEVS_-LipC12 being only 75% of the value obtained for cLipC12. In other words, the presence of the His-tag decreases the catalytic efficiency of LipC12 with *p*NPD as the substrate.

### Activity and stability in organic solvents of His_TEVS_-LipC12 and cLipC12

The use of organic solvents instead of water in biocatalytic processes involving lipases has intensified over the last few decades^9^. Lipases must therefore have both high activity and high stability in organic solvents if they are to be useful biocatalytic tools^27^. Based on the difference in activity found in aqueous media using untagged LipC12 (CSF-rLipC12, cLipC12 and I-CSF-rLipC12) and His-tagged LipC12 (His-LipC12, His_TEVS_-LipC12 and I-His-LipC12), we investigated His_TEVS_-LipC12 and cLipC12 with respect to their *p*NPD-hydrolyzing activity and stability in various concentrations of different polar organic solvents (ethanol, isopropanol, methanol, propanol, acetone, acetonitrile, DMF and DMSO). The activities were expressed as relative activities, in other words, as percentages of the corresponding activity in 50 mM Tris-HCl buffer (pH 7.5) used in the standard reaction.

**Figure 4:**
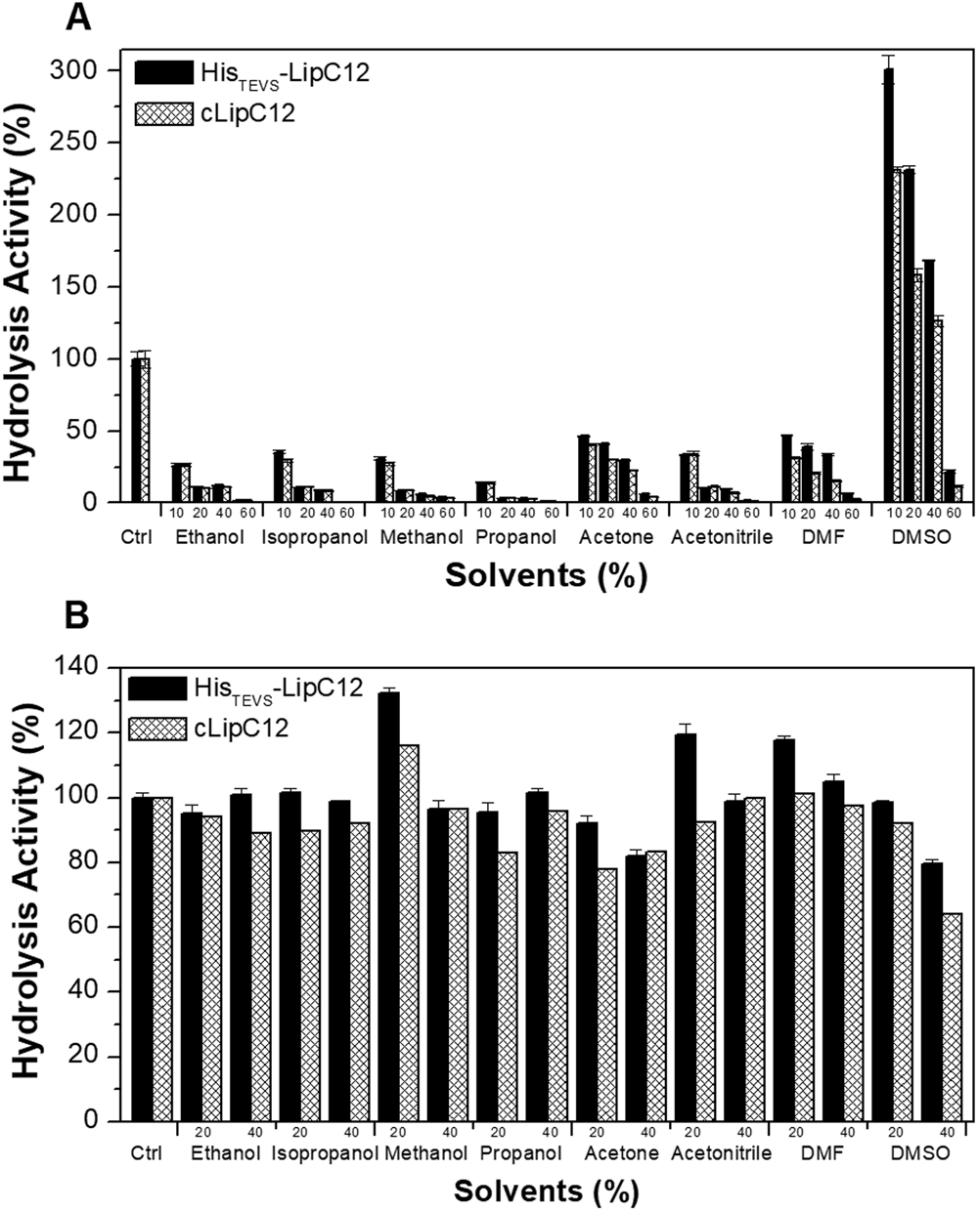
Relative activities (**A**) and stabilities (**B**), compared to the control, of His_TEVS_-LipC12 and cLipC12 in the presence of different concentrations of polar organic solvents. Activities were determined by the spectrophotometric method using the hydrolysis of *p*-nitrophenyl decanoate. The stability in organic solvents was determined after 3 h incubation at 25 °C.

His_TEVS_-LipC12 showed significantly higher activity in the presence of acetone, DMF and DMSO than cLipC12. For both variants of LipC12, the relative activities were lower than 50% for all organic solvents and at all concentrations tested, except for DMSO (Fig. 4A). For this solvent, concentrations up to 40% activated both enzymes, with His_TEVS_-LipC12 being activated to a greater extent than cLipC12. It is well known that polar organic solvents like methanol, ethanol, propanol and butanol inhibit lipase activity^28^. On the other hand, DMSO has been shown to activate lipases several-fold^22,29^. Although the mechanism of this activation has not been elucidated, it has been suggested that DMSO stabilizes the transition state by replacing water in the enzyme-substrate complex^30,31^.

To evaluate the stability in organic solvents, His_TEVS_-LipC12 and cLipC12 were incubated for 3 h at 25 °C in two concentrations (20% and 40% (v/v)) of the polar organic solvents (Fig. 4B). The hydrolytic activity against *p*NPD was then determined under standard conditions (using final enzyme concentrations of 7 nM for His_TEVS_-LipC12 and 5 nM for cLipC12). In most cases, His_TEVS_-LipC12 was more stable than cLipC12 (Fig. 4B). However, in 20% ethanol, 40% methanol, 40% acetone, 40% acetonitrile and 40% DMF, the residual activities of the two enzymes were not significantly different (P > 0.05). The activity of His_TEVS_-LipC12 increased approximately 1.2-fold after incubation in 20% methanol, 20% acetonitrile and 20% DMF. The stability of His_TEVS_-LipC12 in DMSO was not higher than in the other hydrophilic solvents, since its residual activity was 80% after incubation in 40% DMSO (Fig. 4B). Both His_TEVS_-LipC12 and cLipC12 were more stable in polar organic solvents than lipase P21 of *Pseudomonas fluorescens*, which had residual activities below 50% after incubation for 2 h at 30 °C in 3:1 (v/v) solutions of methanol, ethanol, acetone, isopropanol and butanol in water^32^.

### Immobilization of His_TEVS_-LipC12 and cLipC12

Purified His-LipC12 was immobilized on Immobead 150 in previous work^16^. The authors proposed that the His-tag was involved in the covalent bonding with the support. To investigate this question, we immobilized His_TEVS_-LipC12 and cLipC12 onto the same support.

For both His_TEVS_-LipC12 and cLipC12, an immobilization efficiency of 100% was obtained using a protein loading of 10 mg g^−1^. Despite this high immobilization efficiently, the retention of hydrolytic activity in aqueous medium, measured using triolein, was low (0.21% for His_TEVS_-LipC12 and 0.27% for cLipC12). A possible explanation for the poor triolein-hydrolyzing activities of the immobilized preparations is that triacylglycerols like triolein form droplets in aqueous media, with these droplets being bigger than the pores of the support, such that the substrate is only accessible to the enzyme immobilized at the outer surface of the support. This can cause the immobilized lipases to have much lower activities against triacylglycerols than that of the free enzyme or that obtained in organic medium^33,34^.

To confirm covalent immobilization, these immobilized preparations were boiled for 30 min in a solution containing SDS. After SDS-PAGE, in both cases, approximately 8% of the immobilized protein desorbed. This shows that, for both immobilized enzymes, most of the protein was covalently attached to Immobead 150. Since both variants produced the same results, it can be deduced that the His-tag does not favor the covalent binding of LipC12 to Immobead 150.

In accordance with the findings for the variants of LipC12 that had been immobilized from the crude soluble fraction (I-CSF-His-LipC12 and I-CSF-rLipC12), the olive-oil-hydrolyzing activity of I-cLipC12 (123 ± 2 U g^−1^) was higher than that of I-His_TEVS_-LipC12 (54 ± 1 U g^−1^). On the other hand, the presence of the His-tag increased esterification activity for the substrates octanoic acid, lauric acid, palmitic acid and oleic acid (Table 3). At longer reaction times, the performance of I-His_TEVS_-LipC12, with conversions at 9 h above 90%, was also higher than that of I-cLipC12, which gave conversions at 9 h below 68%. To verify the stability of I-His_TEVS_-LipC12 and I-cLipC12 throughout the reaction, the immobilized preparations were recovered after 10 h by filtration, washed with *n*-hexane, dried, and the residual hydrolytic activities against olive oil were measured. The losses of olive-oil-hydrolyzing activity were about 50% for I-His_TEVS_-LipC12 and 74% for I-cLipC12, showing that the removal of the His-tag decreases the stability of I-cLipC12 in the reaction medium, which initially contains ethanol and *n*-hexane, with ethyl caprylate, ethyl laurate, ethyl palmitate or ethyl oleate being produced during the reactions. This result is in agreement with the lower stability found for cLipC12 when it was incubated in polar organic solvents (Fig. 4B).

**Table 3:** Esterification activity of I-cLipC12 and I-His_TEVS_-LipC12 with fatty acids of different chain lengths and ethanol as the alcohol.

Fatty acid	I-cLipC12		I-His_TEVS_LipC12	
	Activity (U g^−1^)	Conversion (%)^*^	Activity (U g^−1^)	Conversion (%)^*^
Octanoic acid (C8:0)	1.51 ± 0.03	26.1 ± 0.5	5.90 ± 0.03	90.8 ± 1.7
Lauric acid (C12:0)	1.65 ± 0.08	26.4 ± 2.5	6.84 ± 0.15	100.0 ± 0.7
Palmitic acid (C16:0)	2.32 ± 0.19	42.2 ± 2.2	7.88 ± 0.22	97.5 ± 0.2
Oleic acid (C18:1)	5.26 ± 0.03	67.9 ± 0.3	9.21 ± 0.01	96.8 ± 0.4

*Conversion of free fatty acids at 9 h of the esterification reaction.

Values represent the mean of triplicates ± standard deviation of the mean.

## Discussion

Our work represents the first time that a His-tagged lipase (I-His_TEVS_-LipC12) has been shown to have lower hydrolytic activity but higher esterification activity, when compared to a similar recombinant enzyme without the His-tag (I-cLipC12). Since lipases are used both for hydrolysis and synthesis reactions, this opens up the possibility of removing the His-tag or not, after expression, in order to tailor the enzyme for the specific application that is intended.

Various previous studies have reported the effects of affinity tags on hydrolytic and esterification activities, as we discuss below. In our comparisons with the literature, we will use the prefix “wt” to indicate a wild type lipase, indicating that the lipase was not produced using molecular biology techniques. The prefix “r” will be used to indicate a recombinant lipase without a His-tag. Since His-tag proteins are recombinant proteins, where possible, we will compare the His-tagged lipases with similar recombinant lipases. Where the authors did not give results for a recombinant lipase, we will undertake comparisons with the wild type lipase.

The most important result of our work is that a His-tag on LipC12 negatively affected hydrolytic activity but promoted esterification activity. His-tagged variants of LipC12 consistently gave lower hydrolytic activities in aqueous media. Both free (CSF-His-LipC12 and His_TEVS_-LipC12) and immobilized (I-CSF-His-LipC12 and I-His_TEVS_-LipC12) enzymes had lower specific olive-oil-hydrolyzing activities than their non-His-tagged counterparts in free (CSF-rLipC12 and cLipC12) and immobilized (I-CSF-rLipC12 and I-cLipC12) forms. Likewise, for the hydrolysis of *p*NPD, the value of *k*_cat_/*K*_M_ for His_TEVS_-LipC12 was 75% of the value obtained for cLipC12. The situation was different for esterification of oleic acid with ethanol in *n*-hexane. In this case, I-CSF-His-LipC12 and I-CSF-rLipC12 had similar activities, but, on the other hand, I-His_TEVS_-LipC12 had both a significantly higher activity and a better stability in the reaction medium than did I-cLipC12.

We suggest that these results might be explained as follows: the His-tag is highly flexible in aqueous medium and some of its movements may temporarily block access of substrates to the active site. There are two possibilities: the flexible His-tag may interfere with the opening of the lid or it might interact with the active site of other lipases in the reaction medium, as has been shown by Majoreck *et al*.^3^. To investigate the second possibility, we added two non-ionic detergents (Triton X-100 and Nonidet P-40) to reaction media during the hydrolysis of *p*NPD, with the aim of separating aggregates of enzyme molecules. For both detergents, we used concentrations both above and below the critical micellar concentration (CMC), which is 0.24 mM for Triton X-100 and 0.08 mM for Nonidet P-40. The effects of the detergents on the two lipases were similar, although the activity of the LipC12 variant without the His-tag (cLipC12) was higher than that of the variant with the His-tag (His_TEVS_-LipC12) at all detergent concentrations (Supplementary Figure S7). This result suggests that there is no interaction between different lipase molecules and, therefore, that the second possibility does not occur.

On the other hand, proteins tend to be less flexible in hydrophobic organic solvents of relatively high log P values, such as *n*-hexane^35^, which we used in the esterification reactions. In this medium, the His-tag would also be less flexible, for example, it might be trapped within the solvation layer, and therefore unable to interfere with the access of substrates to the active site. NMR analysis or molecular dynamics studies of LipC12, with and without the His-tag in aqueous solution and in the presence of organic solvents, would be needed to confirm these suggestions. We recognize that our explanation does not explain why I-His_TEVS_-LipC12 had a significantly higher esterification activity than I-cLipC12. However, His-tags can have subtle effects on protein structure^6^. For example, although a His-tag did not affect the overall structure of myoglobin, it did alter the picosecond-scale dynamic of the protein; this change can affect the recognition of ligands^36^.

The effects of His-tags on lipases have been most studied with respect to their effects on hydrolytic activities (Table 4). Various authors have compared specific activities of lipases with and without His-tags, using several different substrates. Results are variable, with His-tags having been reported to have no effect or to cause decreases and increases of specific hydrolytic activity. In our case, the His-tag decreased the specific olive-oil-hydrolyzing activities (values were 2555 U mg^−1^ for His_TEVS_-LipC12 and 4590 U mg^−1^ for cLipC12). Decreases in specific hydrolytic activity due to the His-tag have been reported previously. For example, for *Staphylococcus aureus* lipase 3 (SAL3), the specific activity of His-SAL3 against tributyrin (4000 U mg^−1^) was lower than that of the recombinant untagged enzyme (r-SAL3, 4500 U mg^−1^)^14^. Likewise, for the hydrolysis of tributyrin (TC4) and olive oil by the lipase of *S. xylosus*, His-SXL had lower specific activities (1500 and 850 U mg^−1^, respectively, for TC4 and olive oil) than did r-SXL (2000 and 1400 U mg^−1^, respectively, for TC4 and olive oil)^37^. However, in some cases, the specific hydrolytic activity was higher with the His-tag. For example, in the case of SAL3, the specific activity against olive oil of His-SAL3 (1300 U mg^−1^) was higher than that of r-SAL3 (1100 U mg^−1^). Similar results were obtained for the hydrolysis of TC4 and olive oil by the lipase of *S. simulans* (SSL). The specific activities of His-SSL against both these substrates (980 and 940 U mg^−1^, respectively, for TC4 and olive oil) were higher than those of r-SSL (850 U mg^−1^, for both TC4 and olive oil)^37^. In some cases, the His-tag caused no significant difference in specific hydrolytic activities. This has been reported for the hydrolysis of *p*NPB, *p*NPL and carboxyfluorescein diacetate by Lipase B of *Candida antarctica* (CALB)^38^, for the hydrolysis of olive oil and TC4 by turkey pancreatic lipase (TPL)^39^ and for the hydrolysis of TC4, trioctanoin (TC8) and olive oil by the lipase of *Fusarium solani*^40^ (Table 4).

**Table 4:** Effects of His-tags on lipases.

Lipase	Tag	Aspect studied	Key results for His-tagged protein relative to untagged protein	Article
Metagenomic lipase LipC12, expressed in *Escherichia coli*	N-terminal His-tag (Six histidine residues)	Specific hydrolytic activities against TC4, TC8, TC18, olive oil, *p*NPD	Lower	This work
		Hydrolytic activities against *p*NPD in presence of polar organic solvents	Higher in acetone, DMF and DMSO	
		Stability in polar organic solvents	Higher in methanol, acetonitrile and DMF	
		Kinetic parameters for *p*NPD hydrolysis	*K*_M_ higher and *k*_cat_/*K*_M_ lower	
		Synthesis of ethyl oleate over 10 h with immobilized lipase	Higher	
*Staphylococcus xylosus* Lipase (SXL)	N-terminal His-tag (Six histidine residues)	Kinetic parameters for hydrolysis of TC4 and TC8	*K*_M_ and *k*_cat_ lower; *k*_cat_ /*K*_M_ higher for TC4, but not affected for TC8	Mosbah *et al*.^12^
Lipase B of *Candida antarctica* (CALB) expressed in *Escherichia coli*	N-terminal FLAG and C-terminal His-tag	Specific hydrolytic activities against *p*NPB, *p*NPL and CFDA	No difference	Blank *et al*.^38^
		Kinetic parameters for hydrolysis of *p*NPB and *p*NPL	No difference	
Lipase B of *Candida antarctica* (CALB)	Fusion proteins (Mdh, PotD, SlyD, GST, MBP) on the N-terminal and C-terminal His-tag	Kinetic parameters for hydrolysis of *p*PNB	*K*_M_, *k*_cat_ and *k*_cat_/*K*_M_ lower for all tags	Seo *et al*.^41^
*Staphylococcus aureus* Lipase (SAL)	N-terminal His-tag (Six histidine residues)	Specific hydrolytic activities	Lower in tributyrin, higher in olive oil	Horchani *et al*.^14^
		Kinetic parameters for hydrolysis of TC4	*k*_cat_/*K*_M_ lower	
		Immobilization on CaCO_3_	Immobilization efficiency lower	
		Synthesis of butyl oleate with immobilized lipase over 24 h	Lower conversion of oleic acid to ester	
		Stability in organic solvents	Lower stability in organic solvents (*n*-hexane, *tert*-butanol and chloroform) for both free and immobilized forms	
Lipases of *Staphylococcus simulans* (SSL & r-SSL), *S. xylosus* (SXL & r-SXL) and *S. aureus* (SAL & r-SAL)	N-terminal His-tag (Six histidine residues)	Specific hydrolytic activities	Lower with TC4 and with olive oil for His-SXL, higher with TC4 and with olive oil for His-SSL	Horchani *et al*.^37^
Turkey pancreatic lipase (TPL)	N-terminal His-tag (Six histidine residues	Specific hydrolytic activities against olive oil and TC4	No significant difference	Bou Ali *et al*.^39^
		Kinetic parameters for hydrolysis of olive oil and TC4	No significant difference in *k*_cat_ and *k*_cat_/*K*_M_	
*Yarrowia lipolytica* Lipase YlLip11	N-terminal His-tag (Six histidine residues)	Activity against *p*NPP	Tagged proteins were produced, but inactive	Kumari *et al*.^48^
*Fusarium solani* lipase expressed in *Pichia pastoris*	N-terminal His-tag (Six histidine residues)	Specific activities against TC4, TC8, olive oil, phospholipids (egg-yolk PC) and galactolipids (1,2-octanoyl-3-O-α-D-galactosyl-sn-glycerol (DiC8-MGDG))	No difference	Jallouli *et al*.^40^

Some authors have analyzed the effect of the His-tag on kinetic parameters (Table 4). Again, the results are quite variable. In our case, the His-tag did not have any effect on *k*_cat_ for hydrolysis of *p*-nitrophenyl decanoate, but increased *K*_M_ 1.3-fold, such that *k*_cat_/*K*_M_ for His_TEVS_-LipC12 was 75% of the value obtained for cLipC12. In other words, the presence of the His-tag decreases the catalytic efficiency of LipC12 with *p*NPD as the substrate. Decreases in *k*_cat_/*K*_M_ with the presence of the His-tag have been reported previously: for SAL3 with TC4 as the substrate, *k*_cat_/*K*_M_ for His-SAL3 was 74% of the value obtained with r-SAL3^14^; for SXL with TC8 as the substrate, *k*_cat_/*K*_M_ was 80% of the value obtained for wt-SXL^12^ and for various variants of CALB with a fusion protein at the N-terminus and a His-tag at the C-terminus, the His-tagged proteins had values of *k*_cat_/*K*_M_ for the hydrolysis of *p*-nitrophenyl butyrate (*p*NPB) that were from 85 to 98% of the value for the unmodified CALB^41^. Increases in *k*_cat_/*K*_M_ have also been reported for His-tagged lipases with TC8 as the substrate: for SAL3, the value of *k*_cat_/*K*_M_ for His-SAL3 was 1.5-fold higher than that for r-SAL3^14^ while for SXL, the value of *k*_cat_/*K*_M_ for His-SXL was 1.2-fold greater than that for wt-SXL^12^. There are also some reports of no significant differences in the kinetic parameters between the His-tagged and non-tagged forms: this was the case for a recombinant CALB, for both *p*NPB and *p*-nitrophenol laurate (*p*NPL) as substrates^38^ and for TPL with TC4 as the substrate^39^.

Significantly, our work represents the first report of a His-tag leading to an increase in esterification activity of a lipase: for the synthesis of ethyl-oleate in *n*-hexane, the specific activity of I-His_TEVS_-LipC12 (9.21 ± 0.01 U g^−1^) was significantly higher than that of I-cLipC12 (5.26 ± 0.03 U g^−1^). The only previous study of the effects of His-tags on esterification activity, undertaken by Horchani *et al*.^14^, involved the synthesis of butyl oleate in *n*-hexane with three immobilized variants of *S. aureus* lipase 3: I-wt-SAL3, I-r-SAL3 and I-His-SAL3. Based on the conversions at the first sampling time of that study, 4 h, one can infer that r-SAL3 and His-SAL3 gave similar initial esterification rates, which were much lower than that obtained with I-wt-SAL3. Horchani *et al*.^14^ followed the reaction profiles over 24 h, with I-wt-SAL3 giving a conversion of around 70% in 20 h and both r-SAL3 and His-SAL3 giving much lower conversions at this time, of around 20%^14^. The key difference from our work is that, in their case, the presence of the His-tag did not improve the esterification performance of SAL3.

Our study is also the first study of the effect of a His-tag on the stability of a lipase in polar organic solvents. Our key result was that, after a 3-h incubation, His_TEVS_-LipC12 had activities around 1.2-fold greater than cLipC12 in the cases of 20% methanol, 20% acetonitrile, 20% DMF and 40% DMSO. The only previous direct study of the effect of a His-tag on the stability of a lipase in solvents was undertaken by Horchani *et al*.^14^. They incubated immobilized variants of SAL3 in non-polar solvents, including *n*-hexane, the solvent that they had used in their esterification studies. After a 24-h incubation in *n*-hexane, both free and immobilized wt-SAL3 and r-SAL3 retained 100% activity, while free and immobilized His-SAL3 had lower residual activities, of 70 and 80%, respectively. The key difference from our work is that, in the case of SAL3, the presence of the His-tag decreased stability in the solvents tested, although it should be noted than *n*-hexane is much less polar than the solvents that we used in our stability studies. The results of Horchani *et al*.^14^ suggest, indirectly, that both I-r-SAL3 and I-His-SAL3 are denatured by polar solvents. The explanation is as follows. The low final conversions that they obtained at 20 h must have been caused by denaturation, as the reaction profiles had leveled out. Since both I-r-SAL3 and I-His-SAL3 had given residual activities above 80% after 24-h incubations in *n*-hexane in their stability study, the loss of activity of these preparations in the esterification reaction was not due to the presence of *n*-hexane. It is possible to infer, then, that both I-r-SAL3 and I-His-SAL3 were denatured by one of the reaction species involved in the synthesis of butyl oleate, possibly the butanol, with the presence of the His-tag not improving stability. Contrary to these results of Horchani *et al*.^14^, in our case the presence of the His-tag did improve stability within the esterification reaction medium, with the residual activity after 10 h being 54% for I-His_TEVS_-LipC12 against only 24% for I-cLipC12.

In the case of immobilization of lipases, only one previous study has addressed the effect of His-tags. In this study, Horchani *et al*.^14^ immobilized wt-SAL3, r-SAL3 and His-SAL3 on CaCO_3_. The immobilization yield was high for wt-SAL3 (79%). It was significantly lower for r-SAL3 (22%) and much lower still for His-SAL3 (2.5%), suggesting that the His-tag did interfere somehow with immobilization. This effect of the His-tag is different from the effect in the current work, in which the His-tag did not affect immobilization.

Our study demonstrates that the His-tag decreases the specific hydrolytic activities of both the free and immobilized forms of LipC12 against triacylglycerols. However, in several polar solvents, both the *p*NPD-hydrolyzing activity and the stability were favored by the presence of the His-tag. Importantly, our study represents the first time that a His-tag has increased esterification action. Our work gives insights into tailoring lipases for specific applications: for hydrolysis reactions, the His-tag should be removed from LipC12, while for esterification reactions, it should be retained.

## Methods

### Bacterial strains, plasmids and protein expression

LipC12 is a lipase isolated by Glogauer *et al*.^15^ from a metagenomic library prepared from DNA extracted from fat-contaminated soil of a waste treatment plant. The gene *lipC12* was cloned into pET28a(+) (this plasmid is referred to as pET28lipC12) to produce an N-terminal His-tagged protein (denominated His-LipC12) that has a theoretical molecular weight of 33.4 kDa. The *lipC12* gene was also cloned into pET29a(+). This was done by digesting pET28lipC12 with *NdeI* and *BamHI* and ligating the *lipC12* coding region into the pET29a vector (Novagen, Hertfordshire, United Kingdom), which had been previously digested with the same restriction enzymes. The resulting plasmid was called pET29lipC12. It was used to produce recombinant LipC12 without a His-tag (denominated rLipC12).

Additionally, the *lipC12* gene was cloned into the pTEV5 vector to obtain the plasmid pTEV5lipC12. This was done by digesting pET28lipC12 with *NdeI* and *BamHI* and ligating the *lipC12* coding region into the pTEV5 vector, which had been previously digested with the same restriction enzymes. The LipC12 produced in cells harboring pTEV5lipC12 is named His_TEVS_-LipC12 and contains a His-tag at the N-terminal and a TEV protease site (TEVS) to allow His-tag cleavage with TEV. After TEV cleavage, the protein is named cLipC12, which is an un-tagged LipC12 that contains 4 extra amino acids (GASH).

His-LipC12, rLipC12, His_TEVS_-LipC12 and TEV protease were overexpressed in *Escherichia coli* strains harboring the plasmids listed in Supplementary Table S2. The *E. coli* strains and expression conditions are also described in Supplementary Table S2. The overexpression and cell disruption by ultrasonication were done as previously described^15,16^.

### Protein purification

His_TEVS_-LipC12 was purified using a 5-mL HiTrap Chelating column (GE Healthcare, Uppsala, Sweden) preequilibrated with buffer A (20 mM Tris-HCl pH 7.5, 150 mM NaCl, 10% (v/v) glycerol and 20 mM imidazole). The protein was eluted stepwise, using increasing concentrations (from 50 to 500 mM) of imidazole in buffer A, with 2 column volumes of buffer being passed at each concentration. Fractions with purity over 95%, as judged by SDS-PAGE (Supplementary Fig. S1), were pooled. Imidazole was removed in a HiTrap Desalting column (GE Healthcare, Uppsala, Sweden) using a 20-mM Tris-HCl buffer at pH 7.5 that contained 150 mM NaCl and 5% (v/v) glycerol. TEV protease was purified as previously described^42^.

### Cleavage of the His-tag with TEV protease

Purified His_TEVS_-LipC12 and TEV protease were mixed in 2 mL of buffer (20 mM Tris-HCl pH 7.5 and 150 mM NaCl), in a mass ratio of His_TEVS_-LipC12 to TEV protease of 10:1. The mixture was maintained at 8 °C, 90 rpm for 20 h. It was then passed through a 5-mL HiTrap Chelating column (GE Healthcare). cLipC12 was eluted with 100 mM imidazole and TEV protease was eluted with 300 mM imidazole. Imidazole was removed from the LipC12 preparation in a desalting column, using a 20 mM Tris-HCl buffer at pH 7.5 that contained 150 mM NaCl and 5% (v/v) glycerol.

The efficiency of cleavage of the His-tag was confirmed by 12% (w/v) SDS-PAGE followed by western blotting and incubation with the anti-His antibody Ni-NTA HPR (Qiagen, Hilden, Germany).

### Titrimetric determination of triacylglycerol-hydrolyzing activity

The triacylglycerol-hydrolyzing activities of the soluble fractions of crude extracts and of purified lipase preparations were determined by the titrimetric method using an automatic titrator (Metrohm 718 STAT Titrino potentiometric titrator, Metrohm, Herisau, Switzerland) with 0.05 M NaOH during 5 min. The conditions have been previously described^16,43^. The reactions were performed at 30 °C using 20 mL of emulsion at pH 7.0. One unit of hydrolytic activity in aqueous medium corresponds to the release of 1 µmol of fatty acid per min, under the assay conditions.

### Spectrophotometric determination of *p*NPD-hydrolyzing activity

The *p*NPD-hydrolyzing activities of the lipases in aqueous solution were determined by monitoring the release of *p*-nitrophenol from *p*-nitrophenyl decanoate (Sigma, St. Louis, USA) at 410 nm at 25 °C in an iMark™ Microplate Absorbance Reader (Bio-Rad, Hercules, USA).

The standard assay conditions have been previously described^15^. The final volume of the reaction mixture was 250 μL. It contained 50 mM Tris-HCl buffer, pH 7.5, 1 mM CaCl_2_, 0.6% (v/v) Triton X-100, 1 mM *p*NPD, 4% (v/v) isopropanol, 1% (v/v) acetonitrile and an enzyme concentration of 172 nM when His_TEVS_-LipC12 was assayed and of 123 nM when LipC12 was assayed. One unit of lipase activity was defined as 1 μmol of *p*-nitrophenol produced per min. Linear regressions to determine initial reaction velocities were performed with Microsoft Excel.

The *p*NPD-hydrolyzing activities of the lipases in the presence of polar organic solvents were determined at concentrations varying from 10 to 60% (v/v) of the solvent (ethanol, isopropanol, methanol, propanol, acetone, acetonitrile, dimethylformamide (DMF) or dimethylsulfoxide (DMSO)) in water. The activity was determined in a 96-well plate by monitoring the release of *p*-nitrophenol from *p*NPD, according to standard assay conditions with the addition of the stated solvent concentrations.

The stability of the lipases was determined by measuring the residual activity after incubation (at final concentrations of 1.72 μM for His_TEVS_-LipC12 and 1.23 μM for LipC12) for 3 h at 25 °C in the same polar organic solvents described for activity. The enzymes were then diluted in 50-mM Tris-HCl buffer at pH 7.5, 150 mM NaCl and 2-mM CaCl_2_ in the reaction media to reach the protein concentrations of 172 nM and 123 nM used in the standard assay of *p*NPD-hydrolyzing activities.

### Determination of kinetic parameters

The kinetic parameters were determined using concentrations of *p*NPD ranging from 0.1 to 3.5 mM and 10 μL of enzyme (enzyme concentration in the reaction medium of 6.9 μM for His_TEVS_-LipC12 and 4.9 μM for LipC12). The reaction was followed for 5 min with the absorbance at 410 nm being read every 30 s. The values of *K*_M_ and *k*_cat_ were determined by non-linear regression, in GraphPad Prism 7, of the data of initial velocity versus substrate concentration, using the Michaelis-Menten equation. The program was used to calculate 95% confidence intervals.

### Circular dichroism

CD spectra of proteins were recorded between 250 and 350 nm using a JASCO J-815 spectropolarimeter (Easton, USA) fitted with a Jasco Peltier-type temperature controller (CDF-426S/15). The optical pathlength was 2 mm and the concentration of the protein solution was 5 μM in a pH 7.4 buffer containing 10 mM sodium phosphate and 100 mM NaCl. The spectra were recorded at 25 °C. The percentages of secondary structures (α-helices and β-sheets) were determined with the software K2D3^44^, after conversion of the data to delta epsilon values (Δε), also referred as the molar circular dichroism^21^.

The melting temperatures (Tm) were determined by measuring the CD signal at 222 nm with increasing temperature in a continuous ramp mode (1 °C/2 min). The temperature was increased from 25 to 98 °C. The Tm was determined from the first derivative using GraphPrism 6.0.

### Lipase immobilization

The covalent immobilization of LipC12 preparations was performed using Immobead 150 (Sigma–Aldrich, St. Louis, USA) according to Madalozzo *et al*.^16^. The immobilization kinetics of crude soluble fractions containing His-LipC12 and rLipC12 were determined with protein loadings of 200 mg g^−1^ during 8-h incubations at 4 °C in an orbital shaker at 150 rpm. Purified His_TEVS_-LipC12 and cLipC12 were also immobilized on Immobead 150 with protein loadings of 10 mg g^−1^, with incubation for 6 h at 4 °C in an orbital shaker at 150 rpm.

A desorption test was done after immobilization. Twenty mg of the support was added to 200 μL of a 2% (m/v) SDS solution. The mixture was heated at 100 °C for 30 min. After that, samples were loaded onto a 12% SDS-PAGE gel for analysis. The percentage of desorption was calculated based on the protein contents of the samples (which were estimated using the protein standards supplied by the manufacturer, GE Healthcare).

### Determination of the esterification activity

The esterification activities of the immobilized preparations of LipC12 were determined based on ethyl-oleate synthesis^16^. The reactions were carried out in 25-mL Erlenmeyer flasks, with 5 mL of reaction medium (210 mmol L^−1^ ethanol and 70 mmol L^−1^ oleic acid in *n*-hexane). The flasks were placed in an orbital shaker at 40 °C and 250 rpm. The reaction was started with the addition of 110 mg of the immobilized enzyme. At each sampling time, a 100-µL sample was collected and analyzed to quantify free fatty acids by the colorimetric Lowry-Tinsley method^45^. One unit of lipase esterification activity (U) corresponds to the consumption of 1 μmol of fatty acid per min, under the assay conditions.

### Protein analysis

The molecular weights of the proteins were determined using the ProtParam tool of the online program Expasy. Protein electrophoresis was carried out by 12% SDS-PAGE^46^ and gels were stained with Coomassie Blue. Densitometry analysis of the protein bands from the SDS-PAGE gel was performed using the LabWorks Software Version 4.0 (UVP BioImaging Systems, Upland, CA). Protein concentrations were determined by the Bradford assay using bovine serum albumin as the standard^47^.

### Statistical analysis

For all assays, the standard deviation of the mean was calculated. The two-tailed unpaired *t* test was used to compare activities and stability values of LipC12 variants with and without His-tags. Differences were evaluated for significance at the 5% level (i.e. P < 0.05). The difference of the kinetic parameters was evaluated based on 95% confidence intervals (profile-likelihood).

### Data availability

The datasets generated and analyzed during the current study are available from the corresponding author on reasonable request.

## Electronic supplementary material

Supplementary Information
