# Supplementary material for: Tailoring recombinant lipases: keeping the His-tag favors esterification reactions, removing it favors hydrolysis reactions
Source: Sci Rep. 2018 Jul 3;8:10000. doi: 10.1038/s41598-018-27579-8 (PMC6030132; doi:10.1038/s41598-018-27579-8)
Supplement: Supplementary file 1 — Supplementary Information [file 41598_2018_27579_MOESM1_ESM.pdf]

SUPPLEMENTARY MATERIAL

FOR

Tailoring recombinant lipases: keeping the His-tag favors esterification reactions, removing it favors hydrolysis reactions

Janaina Marques de Almeida[1#], Vivian Rotuno Moure[1#], Marcelo Müller-Santos[1], Emanuel Maltempi de Souza[1], Fábio Oliveira Pedrosa[1], David Alexander Mitchell[1] and Nadia Krieger[2*].

[1]Departamento de Bioquímica e Biologia Molecular, Universidade Federal do Paraná, Cx.P. 19046 Centro Politécnico, Curitiba 81531-980, Paraná, Brazil.

[2]Departamento de Química, Universidade Federal do Paraná, Cx.P. 19081 Centro Politécnico, Curitiba 81531-980, Paraná, Brazil.

[*] To whom correspondence should be addressed. E-mail: nkrieger@ufpr.br; Telephone: +55-41-33613470

[#] J.M.A and V.R.M. contributed equally to this work

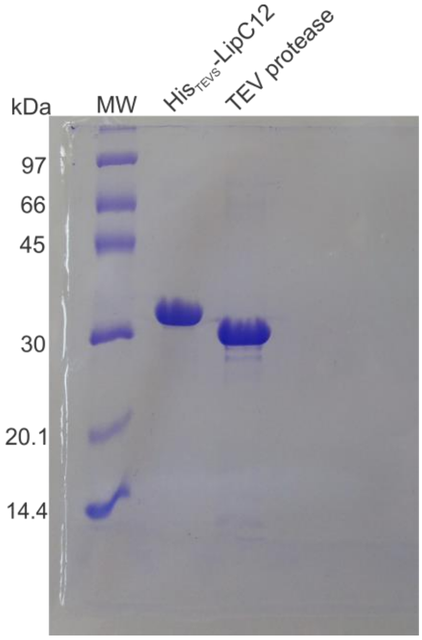

Supplementary Figure S1. Purified fraction of His_{TEVS}-LipC12 and TEV protease. 5 μg of each protein was analyzed on 15% SDS-PAGE and the gel was stained with Coomassie Blue. The molecular weight standard (MW) ladder is shown as kDa. For purification of His_{TEVS}-LipC12, the cellular pellet was resuspended in 30 mL of 20 mM Tris-HCl buffer (pH 7.5) containing 150 mM NaCl, 10% (v/v) glycerol and 20 mM imidazole. The soluble fraction was loaded onto a 5-mL HiTrap chelating column. After washing in buffer containing 50 mM imidazole, protein was eluted with a stepwise gradient of imidazole. The target protein was eluted in 300 mM imidazole. Fractions were pooled and loaded onto a 5-mL desalting column (2×) and eluted in a 20 mM Tris-HCl buffer (pH 7.5) containing 150 mM NaCl and 5% (v/v) glycerol. For purification of TEV protease, the cellular pellet was resuspended in 30 mL of 50 mM sodium phosphate buffer (pH 7.4) containing 300 mM NaCl. The soluble fraction was loaded onto a 1-mL HisTrap column. After washing in buffer containing 20 mM imidazole, protein was eluted with a stepwise gradient of imidazole. TEV was eluted in 250 mM imidazole. Fractions were pooled and loaded onto a 5-mL desalting column (2×) and eluted in a 25 mM sodium phosphate buffer (pH 7.5) containing 200 mM NaCl, 2 mM EDTA, 2 mM DTT and 10% (v/v) glycerol.

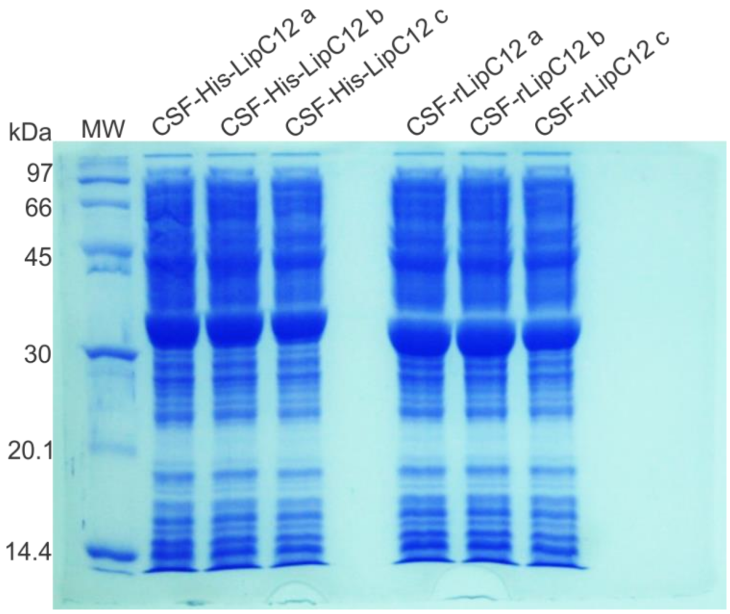

Supplementary Figure S2. Expression of His-LipC12 and rLipC12. Replicates of crude soluble fractions (CSF) containing His-LipC12 and LipC12 (61 and 64 μg of protein, respectively) were analyzed on 12% SDS-PAGE and the gel was stained with Coomassie Blue. The molecular weight standard (MW) is shown as kDa. The target proteins in the gel were analyzed by densitometry using the LabWorks program.

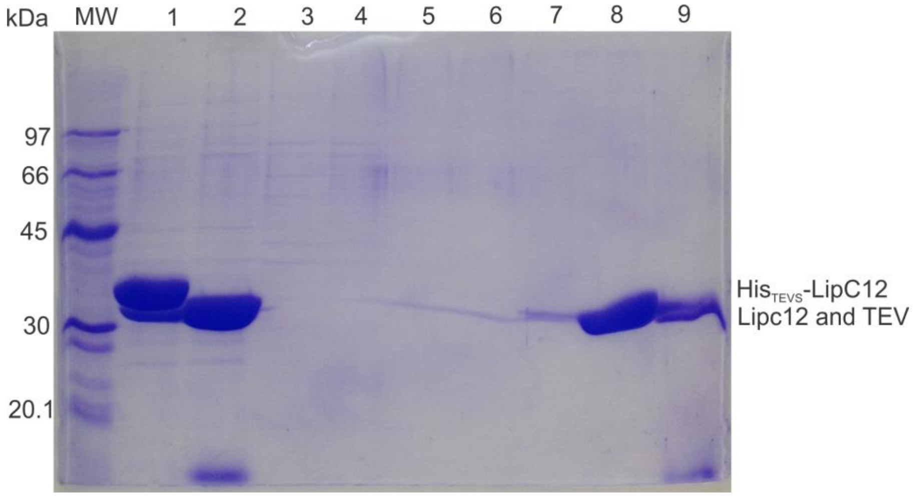

Supplementary Figure S3. Representative profile of His-tag cleavage of LipC12 with TEV. The substrate His$_{TEVS}$-LipC12 was mixed with TEV protease at a substrate:TEV ratio of 10:1. After incubation at 8 °C and 90 rpm for 20 h, the mixture was loaded onto a 5-mL HiTrap Chelating column and eluted with buffers containing 100 and 300 mM imidazole. Lane 1: Reaction at time zero. Lane 2: Reaction at 20 h. Lanes 3-7: elution in buffer without imidazole. Lane 8: elution in buffer containing 100 mM imizadole. Lane 9: elution in buffer containing 300 mM imidazole. The molecular weight standard (MW) is shown as kDa.

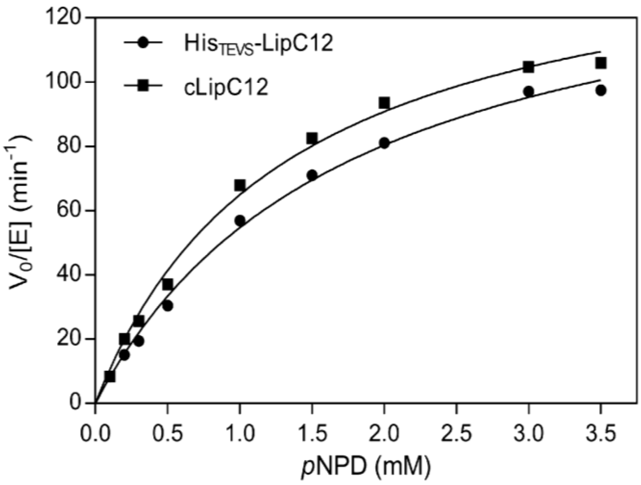

Supplementary Figure S4. Determination of the K_M for *p*NPD using His_{TEVS}-LipC12 and LipC12. The enzyme concentrations were 6.88 nM for HisTEVS-LipC12 and 4.92 nM for LipC12. A control reaction was performed in the absence of enzyme. The K_M, k_{cat} and k_{cat}/K_M values were determined by non-linear regression using GraphPad Prism. V_0 was measured in terms of µmol of product formed per minute.

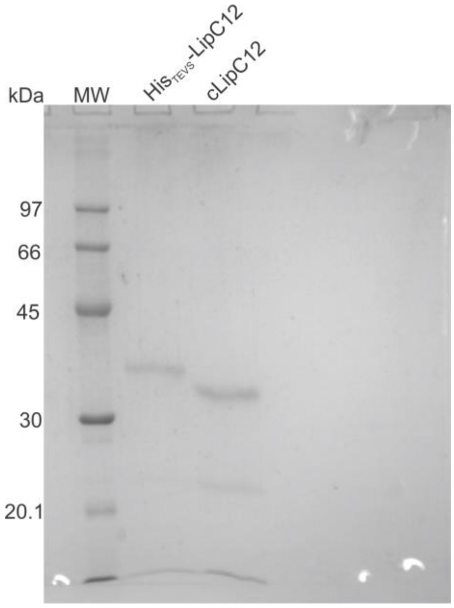

Supplementary Figure S5. Analysis of the desorption of His$_{TEVS}$-LipC12 and LipC12 after immobilization on Immobead 150. 20 mg of each derivative was incubated with 200 µL of a solution containing 2% SDS at 100 °C for 30 min. Samples of 30 µL were loaded onto a 12% SDS-PAGE gel. The gel was stained with Coomassie Blue. Molecular weight standards (MW) are shown as kDa.

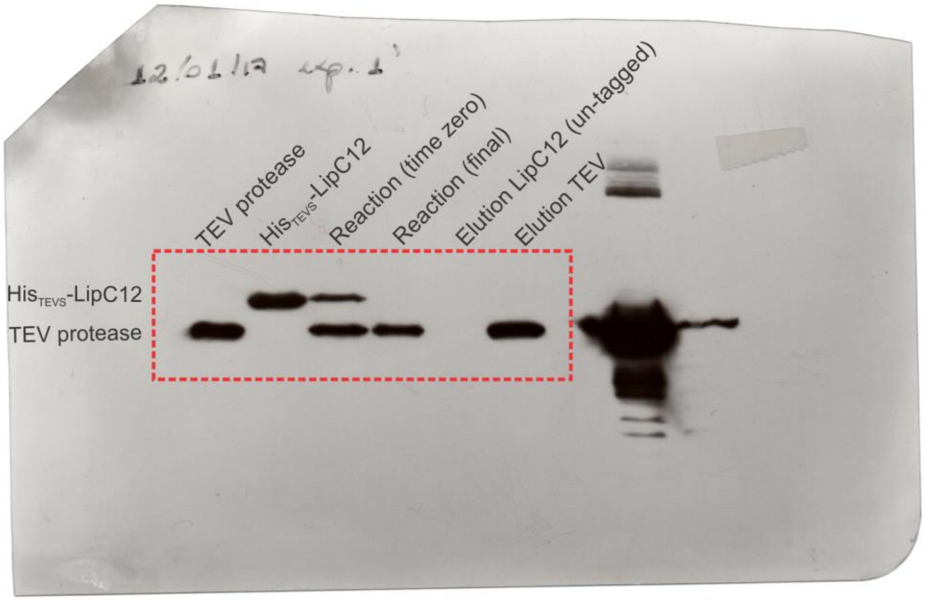

Supplementary Figure S6. Full-length blot of cleavage of His$_{TEVs}$-LipC12 by TEV protease.

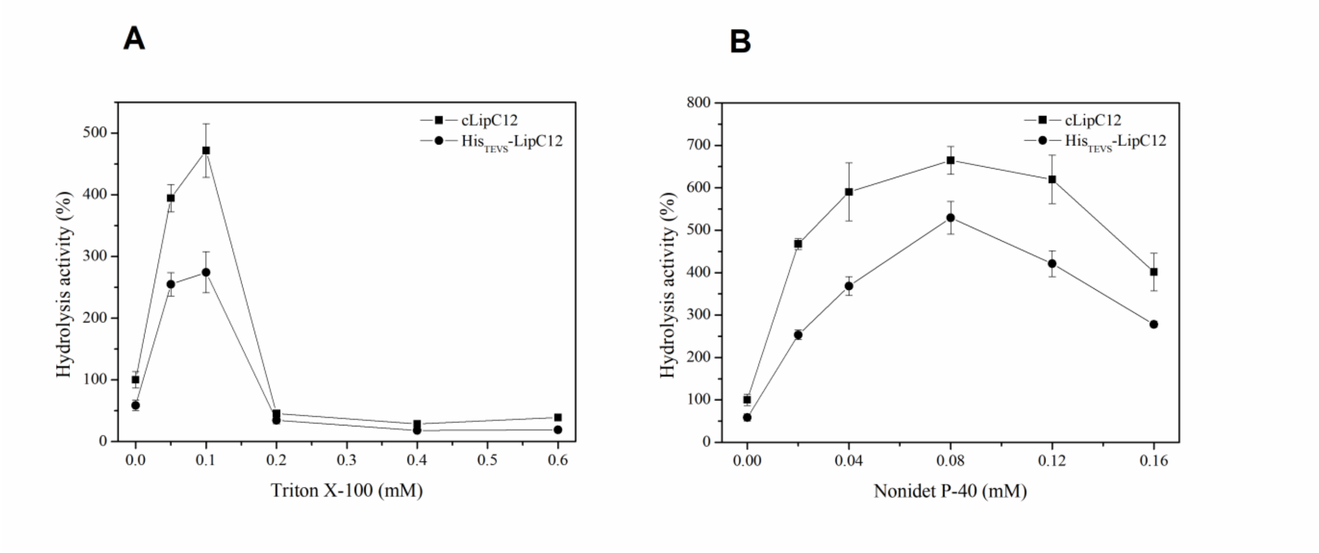

Supplementary Figure S7. (A) Effects of Triton X-100 on the hydrolysis activity of cLipC12 and His_{TEVS}-LipC12. (B) Effects of Nonidet P-40 on the hydrolysis activity of cLipC12 and His_{TEVS}-LipC12. Activities were determined by the spectrophotometric method using the hydrolysis of *p*-nitrophenyl decanoate. The enzyme concentrations were 172 nM for HisTEVS-LipC12 and 123 nM for cLipC12. The assays were done in triplicate. The error bars represent the standard deviations of the means.

Supplementary Table S1. Efficiency of immobilization of His-LipC12 and rLipC12 on Immobead 150.

Time (h)	His-LipC12 (%)	rLipC12 (%)
0	0 ± 0	0 ± 0
2	63 ± 3	66 ± 1
3	68 ± 2	73 ± 1
6	77 ± 1	78 ± 1
8	81 ± 1	81 ± 1

Activities were measured by the titration method using an automatic titrator pHStat and olive oil as the substrate. The values are the mean ± the standard deviation of the mean.

Supplementary Table S2. Plasmids, *Escherichia coli* strains used for protein expression and expression conditions.

Plasmid	Genotype	Reference	*E. coli* strains	Expression conditions
pET28lipC12	Km^r (pET28a). Expresses His-LipC12	Glogauer *et al.*[15]	BL21 (DE3)	0.5 mM IPTG at 18 °C for 16 h
pET29lipC12	Km^r (pET29a). Expresses rLipC12	This work	BL21 (DE3)	0.5 mM IPTG at 18 °C for 16 h
pTEV5lipC12	Amp^r (pTEV5). Expresses His_{TEVS}-LipC12	This work	BL21 (DE3)	0.4 mM IPTG at 16 °C for 16 h
$pTEV_{SH}$	Amp^r (pTH24). Expresses TEV_{SH} protease	van den Berg *et al.*[42]	Rosetta (DE3) pLysS	1 mM IPTG at 20 °C for 22 h
